# Degradation of Organophosphorus and Pyrethroid Insecticides in Beverages: Implications for Risk Assessment

**DOI:** 10.3390/toxics6010011

**Published:** 2018-02-02

**Authors:** Samantha A. Radford, Parinya Panuwet, Ronald E. Hunter, Dana Boyd Barr, P. Barry Ryan

**Affiliations:** 1 Department of Chemistry, Emory University, Atlanta, GA 30322, USA; bryan@emory.edu; 2 Laboratory for Exposure Assessment and Development for Environmental Research, Department of Environmental Health, Rollins School of Public Health, Emory University, Atlanta, GA 30322, USA; parinya.panuwet@emory.edu (P.P.); ronald.e.hunter@gmail.com (R.E.H.J.); dbbarr@emory.edu (D.B.B.); 3 Department of Chemistry, Saint Francis University, Loretto, PA 15940, USA; 4 Analytical Services Lab—Americas, The Coca-Cola Company, Atlanta, GA 30313, USA

**Keywords:** pesticides, degradation, biomarkers of exposure, risk assessment, GC-ECD

## Abstract

Since urinary insecticide metabolites are commonly used as biomarkers of exposure, it is important that we quantify whether insecticides degrade in food and beverages in order to better perform risk assessment. This study was designed to quantify degradation of organophosphorus and pyrethroid insecticides in beverages. Purified water, white grape juice, orange juice, and red wine were fortified with 500 ng/mL diazinon, malathion, chlorpyrifos, permethrin, cyfluthrin, cypermethrin, and deltamethrin, and aliquots were extracted several times over a 15-day storage period at 2.5 °C. Overall, statistically significant loss of at least one insecticide was observed in each matrix, and at least five out of seven insecticides demonstrated a statistically significant loss in all matrices except orange juice. An investigation of an alternative mechanism of insecticide loss—adsorption onto the glass surface of the storage jars—was carried out, which indicated that this mechanism of loss is insignificant. Results of this work suggest that insecticides degrade in these beverages, and this degradation may lead to pre-existing insecticide degradates in the beverages, suggesting that caution should be exercised when using urinary insecticide metabolites to assess exposure and risk.

## 1. Introduction

Organophosphorus (OP) and pyrethroid insecticides are two of the most-used classes of insecticides in the United States, particularly in the agricultural sector [1,2,3]. While insecticide use on crops has contributed to increased crop yields and variety in the American diet over the past 60 years, this usage has also exposed the population to several new environmental toxicants [4].

Exposure to OP and pyrethroid insecticides is often monitored through the quantification of urinary insecticide metabolites [3]. Through the use of these biomarkers of exposure (BOEs), it is assumed that there is a one-to-one correlation between parent insecticide intake and urinary metabolite output. However, there is evidence that this assumption is incorrect. Several studies have shown that pesticides may break down in food or in the environment before a product is consumed. In particular, OPs and pyrethroid insecticides, both containing ester linkages, are susceptible to hydrolysis [5]. Pyrethrins, pyrethroids, and some OP insecticides also are susceptible to photolysis in both soil and water [6,7]. The degradation products formed are often identical to urinary insecticide metabolites [8]. If these metabolites are not further broken down in the human body, their use as BOEs will lead to overestimation of insecticide exposure. Therefore, a better understanding of the rate of degradation of these compounds in foods is needed.

The Joint FAO/WHO Meeting on Pesticide Specifications (JMPR) reports information concerning the degradation of many pesticides [9,10,11,12,13,14]. Most of these studies take place in sterile water, which is not comparable to actual storage conditions for the foods in which these compounds are found. In this study, three OP pesticides (diazinon, malathion, and chlorpyrifos) and four pyrethroids (permethrin, cypermethrin, cyfluthrin, and deltamethrin) were studied for their degradation in water, white grape juice, red wine, and orange juice. After the juices were spiked, they were then held for fifteen days at a typical household refrigerator temperature in amber glass jars for sampling. The loss of parent compound was quantified by gas chromatography with electron capture detection (GC-ECD) using a method that was previously validated using GC-MS/MS with appropriate sensitivity and specificity [15]. In this way, insecticide loss, leading to the presence of hydrolysis degradate production and subsequent BOE overestimation [5], can be quantified. To support that parent loss is through degradation and not other means such as adherence to storage container walls, a separate seven-day study was done with silanized amber glass jars and with vortexing to remove any parent compound off of the glass walls of the container.

## 2. Materials and Methods

### 2.1. Reagents and Materials

Acetonitrile (ACN) (HPLC grade), toluene (Chromasolv grade), methanol (HPLC grade), and glacial acetic acid were purchased from Sigma-Aldrich, Inc. (St. Louis, MO, USA). NaCl (ACS grade) was obtained from EMD (Gibbstown, NJ, USA). Water used was purified in-house to 18.2 MΩ·cm with a Milli-Q^®^ water system (Millipore, Billerica, MA, USA). Supelclean^TM^ ENVI-CARB-II/PSA SPE cartridges (Bed A: 500 mg ENVI-CARB; Bed B: 300 mg primary secondary amine, PSA) were purchased from Sigma-Aldrich, Inc. (Bellefonte, PA, USA). For GC-ECD operation, helium and nitrogen (both zero grade and with 99.999% ultra-high purity) were obtained from Nexair Gases, Inc. (Suwanee, GA, USA).

### 2.2. Standards

Three OP insecticides (diazinon, malathion, and chlorpyrifos) and four pyrethroid insecticides (permethrin, cyfluthrin, cypermethrin, and deltamethrin) were analyzed. The insecticide standards were obtained from the Centers for Disease Control and Prevention (Atlanta, GA, USA) and/or Chem Service, Inc. (West Chester, PA, USA). A stock solution containing all seven insecticides at 10 ppm was prepared in acetonitrile. Stocks were stored in a freezer at −20 °C.

### 2.3. Identification and Quantification of Pesticides

Standards of the seven insecticides in acetonitrile were made in increasing concentrations from 1 ng/L–1000 ng/L and used to create a calibration curve. The calibration curve was created using a power regression curve due to the non-linear response of the ECD. Peaks were manually integrated using ChemStation software (Agilent Technologies, Santa Clara, CA, USA) and data were analyzed using Microsoft Excel 2007 (Microsoft, Redmond, WA, USA).

### 2.4. Fortification and Extraction Protocol

Four matrices were chosen for analysis. Milli-Q (Millipore, Billerica, MA, USA) water, which has been cleaned to a resistance of 18.2 MΩ·cm, was chosen as a simple beverage matrix free from enzymes or other interferences. White grape juice, red wine, and pulp-free orange juice were chosen in order to study beverage matrices with a variety of complexity due to tannins, particulate matter, polyphenols, and other components. Beverages were obtained from a local grocery store. To create sample stocks, 50 mL of water, white grape juice, red wine, and orange juice were each fortified to an initial concentration of 500 ng/mL of a mix of the seven insecticides and stored at 2.5 °C in an amber glass jar in the dark. Extraction and clean-up were performed immediately after insecticide fortification, and then 0.5, 1, 2, 4, 7, 14, and 15 days later (*n* = 3 for each matrix each day). Blanks were also analyzed on day 0 in the same fashion (*n* = 2 per matrix). 

### 2.5. Insecticide Extraction

The extraction procedure is based on that of Hunter et al. [15] with some modifications. A 1.0 mL sample was added to a trace-cleaned 15 mL conical centrifuge tube. Then, ~0.5 g NaCl and 5.0 mL ACN was added to the sample. The sample was vortexed for 3 minutes and centrifuged for 6 min at 1200 rpm. An ENVI-CARB-II/PSA cartridge was preconditioned with 5.0 mL of a 25% (*v*/*v*) solution of toluene in ACN. After preconditioning, 2.0 mL of supernatant from the extracted, centrifuged sample was loaded onto the cartridge. Then, 10.0 mL of the 25% (*v*/*v*) toluene in ACN was eluted through the cartridge, and the eluate was collected in a trace-cleaned 15 mL centrifuge tube. The sample was evaporated using a TurboVap evaporator (Zymark Corporation, Hopkinton, MA, USA) at 20 PSI and 38 °C to near-dryness. The original cartridge was eluted a second time with 25% (*v*/*v*) toluene in ACN and collected in the same tube. The sample was evaporated again at 20 PSI and 38 °C to dryness. Samples were reconstituted with 1.0 mL ACN and stored sealed in the refrigerator at 2.5 °C until analysis, when they were transferred to a GC vial. 

### 2.6. Instrumental Analysis

For GC-ECD separation, a Hewlett-Packard Model 5890A Series II gas chromatograph equipped with an Agilent Technologies electron-capture detector and 7683B Series Injector Autosampler (Santa Clara, CA, USA) was used. A DB-5 column (Agilent Technologies, Inc., Santa Clara, CA, 30 m, 0.25 mm i.d., 0.25 µm film thickness [5% phenyl, 95% dimethylpolysiloxane]) was employed, and a 2 mm i.d. single-taper injection liner was used to prolong column life. Injection volume was 1.0 μL (1:30 split). The helium carrier gas was at a flow rate of 0.88 mL/min, while the nitrogen make up gas flow was 13 mL/min. The injector temperature was 260 °C. The temperature program started at 80 °C and stayed at that temperature for 2 min before being heated linearly by 10 °C/min to a final temperature of 280 °C that was then held for 13 min. The detector temperature was 280 °C. 

### 2.7. Statistical Analysis

Analyte concentrations were determined using the calibration curves described. Linear trend curves relating log concentrations and time in days were determined, and error bars denote standard deviation of the log transform. All confidence intervals were determined from the linear regression results and half-lives were determined using the slope (β_1_) and the relationship *t*_1/2_ = −log(2)/β_1_. The criterion for significance was pre-determined to be a *p*-value < 0.05 for the regression slope. The coefficient of determination (R^2^) represents the fraction of the variance accounted for by the linear model.

### 2.8. Study of Glass/Insecticide Interactions

**Figure 1:**
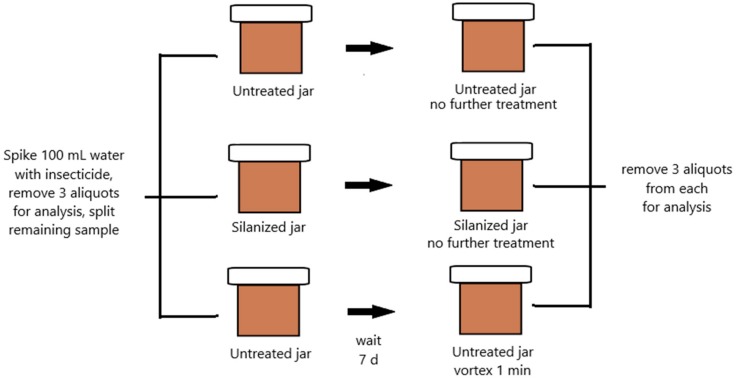
Scheme depicting treatment for study of glass jar/insecticide interactions as a control for insecticide loss due to adsorption.

To confirm that loss of parent compound was due to pesticide degradation rather than adherence to the glass walls of the storage containers, we tested the interaction of insecticides with the glass walls of our containers. Water was chosen as the simplest matrix, free of interfering compounds that could compete with pesticide for jar wall adherence. A 100 mL sample of Milli-Q purified water was fortified with 500 ng/mL of a mix of each of the seven pesticides (Figure 1). In order to test for interactions with the polar glass wall, one amber glass jar (identical to the jars used to store fortified liquids in the above study) was silanized using dimethyldichlorosilane in order to cap any exposed hydroxyl groups. The silanized jar and two other unsilanized jars were used to hold 25.0 mL each of the fortified water. Three 1.0 mL aliquots of fortified water were immediately extracted using the protocol described in the previous section to confirm initial concentration, and the three jars of water were put into a 2.5 °C refrigerator for a week. After the week had passed, three 1.0 mL aliquots each were taken from the silanized jar and from one of the unsilanized jars for extraction. The other jar was vortexed for one minute to test for physical adsorption to the glass wall before three 1.0 mL aliquots were also taken from it for extraction and analysis. The extracted samples were analyzed by GC-ECD using the method described above, and a two-tailed *t*-test was used to determine significant differences in sample concentrations (*p* < 0.05).

## 3. Results

### 3.1. Study of Parent Degradation by GC-ECD

**Figure 2:**
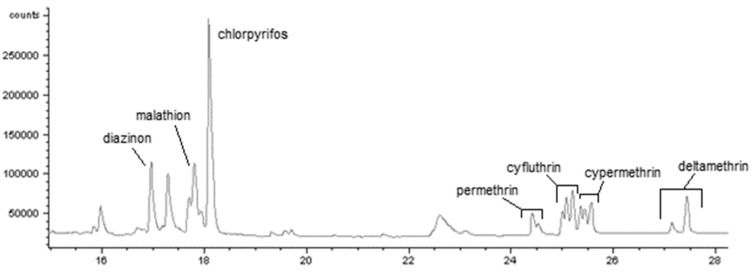
Chromatogram of seven insecticides in orange juice from day 0. Orange juice was chosen to show that even in the most complex matrix, apart from malathion, chromatograms are free from interferences. Pyrethroids show multiplet peaks because of stereoisomerism, including cis/trans isomerism across the cyclopropane ring. Note that the chromatogram reflects the 3:1 isomeric ratio for deltamethrin.

Baseline separation of each insecticide was achieved during analysis (Table 1). Chromatograms were generally clean and did not have interfering peaks with the exception of malathion (Figure 2). The interfering peaks near malathion likely affected quantification, since they caused the baseline to be difficult to determine. No insecticide was found in blank aliquots of samples.

**Figure 3:**
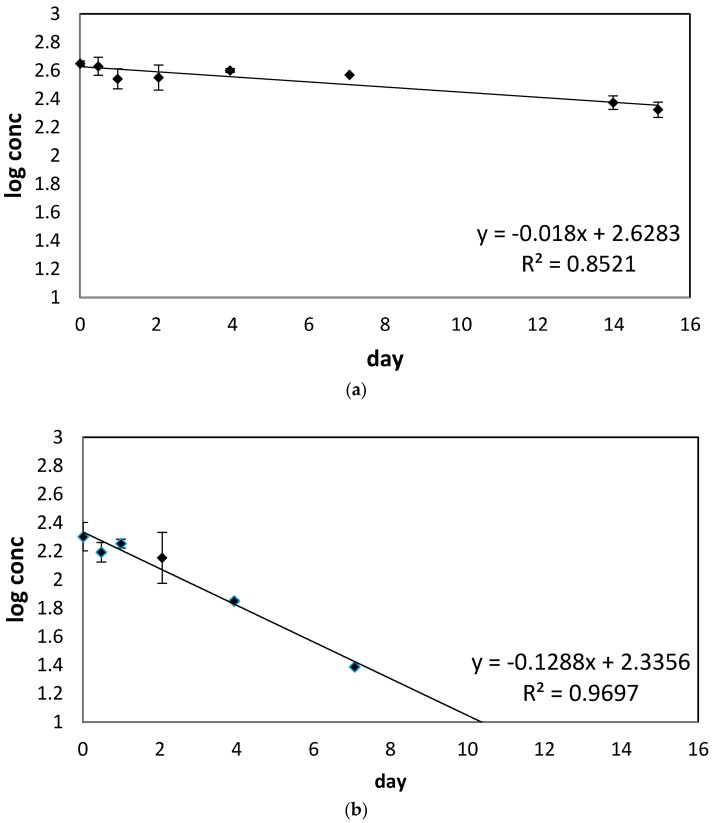
Sample graphical representations of degradation of (**a**) cypermethrin in grape juice and (**b**) diazinon in red wine. Error bars represent standard deviations of each day’s log-transformed concentration.

For each day’s data set, the concentration was log-transformed, and the average of these points was used to determine regression. Because these log-transformed points yielded linear data, first-order kinetics were assumed. Therefore, first-order rate constants and their standard errors are reported in Table 2. The 95% confidence intervals for each rate constant were used to calculate the half-life, with its confidence interval, for each analyte in matrix (Table 2). Figure 3 displays sample graphs of insecticide degradation. Error bars denote standard deviation for the mean of each day’s log-transformed concentration. Data was log-transformed before averaging so that standard deviations of each data point could be accounted for graphically.

### 3.2. Study of Glass/Insecticide Interactions by GC-ECD

**Figure 4:**
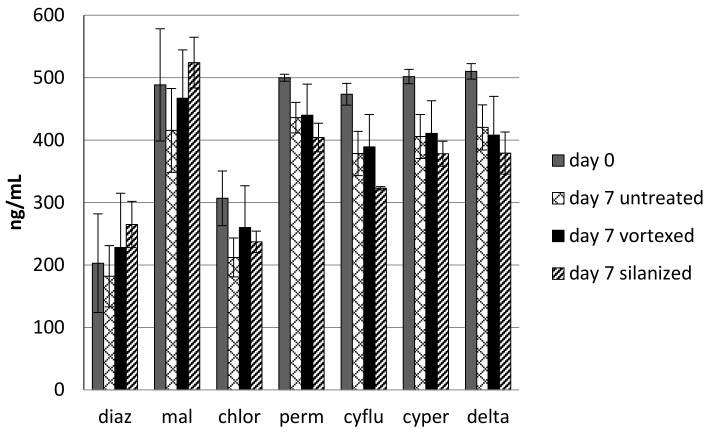
Effect of jar treatment on insecticide degradation. There is no significant difference among day 7 samples except for the silanized cyfluthrin samples, indicating that overall jar adsorption plays an insignificant role in the disappearance of insecticide.

For each insecticide, there was no significant difference in concentration among day 7 samples, except for the silanized/cyfluthrin sample (*p* < 0.05) (Figure 4). Results of the two-tailed *t*-test showed that all pyrethroids showed significant loss (*p* < 0.05) with the untreated jar and the silanized jar, and all pyrethroids except permethrin showed marginally significant loss (*p* < 0.10) in the vortexed jar. As seen in the previous ECD study in water, diazinon and chlorpyrifos showed poor recovery of the initially fortified insecticide on Day 0. Neither diazinon nor malathion showed significant degradation in water over seven days, but the chlorpyrifos in the untreated jar did.

## 4. Discussion

All insecticides showed significant degradation in multiple matrices. The linearity of the log-transformed plots suggests first-order kinetics.

Diazinon degraded especially rapidly (*t*_1/2_ = 2.3–15.2 days depending on matrix). Degradation of diazinon led the concentration of this insecticide to fall below the limit of detection after day 7 in red wine. Although the slope of the regression is non-significant in orange juice, we used the slope to infer a half-life of 12.3 days. The rapid loss of diazinon across matrices is likely due to the fact that while hydrolysis of the other insecticides used in this study is base-catalyzed, diazinon generally degrades more quickly in acidic matrices such as grape juice (pH 2.8–4.2), wine (pH 2.8–4.0), and orange juice (pH 3.6–4.3) [5,16,17,18,19]. This hypothesis is supported by the observation that diazinon showed its longest half-life in water (15.2 days), the only non-acidic matrix tested. The rapid degradation of diazinon may also explain the low recovery of diazinon in each matrix (40.7% in red wine, 69.5% in water). If some of the insecticide degraded in a near-instantaneous fashion upon addition to matrix, inferred recovery would then be low.

Orange juice showed the least degradation of insecticides for all matrices, both by regression significance and half-life. Orange juice has comparable pH to wine and grape juice, so pH differences are likely not responsible for longer half-lives. While half-life and significance are often related, as a larger slope generally is correlated with a greater probability of significance, the variability about the line may reduce significance of data. The greater variability found for orange juice samples may be due to the greater complexity of this matrix, e.g., the analyte may interact with the solid pulp in the juice, since even “pulp-free” juice contains smaller pulp particles. This complexity may also explain the generally longer half-lives for insecticides in orange juice. Pulp may give insecticides surfaces to interact with and adsorb to, creating an intermediate storage mechanism and thereby delaying degradation. As the pulp and other matrix components break down, insecticide is released and then subject to chemical degradation.

Research cited by JMPR notes that deltamethrin is stable to hydrolysis [13]. Our research supports this finding. While deltamethrin in red wine and orange juice displayed a negative regression slope, implying production of insecticide, standard error was greater than the magnitude of the slope, rendering this data statistically insignificant. The standard error was also greater than the magnitude of slope for permethrin in orange juice, again implying insignificant change in pesticide concentration.

Without observation of degradate production, loss of insecticide does not guarantee that these compounds are actually degrading. The loss may be due to some other mechanism. Volatilization is not a suspected mechanism of loss because neither OP nor pyrethroid insecticides display appreciable volatility at 2.5 °C. However, adsorption of the insecticides to the wall of the glass storage container is a more likely alternative mechanism of loss [20]. The results of the insecticide adsorption study suggest that this mechanism is of only minor importance in the observed loss of insecticides from bulk fluids. Although the pesticides from the vortexed jar generally showed a slightly higher concentration of pesticide than the silanized or untreated jars, they also produced the largest standard deviations for the triplicate samples. It is especially noteworthy that there was no statistically significant difference between the concentrations of pesticide in any of the three final day samples in this study, with the exception of cyfluthrin in the silanization trial. Overall, the evidence suggests that there may be some pesticide adsorption by the jar wall, but the amount of insecticide found seems to indicate that loss of insecticides was largely due to some other mechanism.

Loss of analyte would be more rapid and therefore easier to observe at higher temperatures; however, storing juice at a higher temperature for two weeks would not reproduce realistic conditions and would lead to sample spoilage. Therefore, we chose to refrigerate our samples throughout the course of the study. Ideally, studies conducted at the temperature of a refrigerator take place at 4 °C. This study took place at 2.5 °C, because that was the temperature of our refrigerator. This is a limitation of the study, but future studies can be conducted at 4 °C by checking the refrigerator temperature before and throughout the experiment.

We hypothesize that insecticide degradation occurs mainly through hydrolysis. In a similar study to this, we fortified fruit juices to 500 ng/g malathion, chlorpyrifos, and permethrin [21]. Instead of extracting and following concentrations of parent insecticide, three degradates (malathion dicarboxylic acid, MDA, for malathion, 3,5,6-trichloro-2-pyridinol, TCPy, for chlorpyrifos, and 3-phenoxybenzoic acid, 3-PBA, for permethrin) were analyzed instead via LC-MS/MS. MDA and TCPy were both produced in significant concentrations. Production of 3-PBA was not observed in this study, but other work has observed 3-PBA in production [22].

Hydrolysis as the mechanism of insecticide loss is also supported by previous work in which dialkyl phosphates (DAPs) and other hydrolysis pesticide degradates are observed in food. Zhang et al. [23] observed the presence of DAPs in several produce samples known to contain at least one OP insecticide, and 60% of the samples contained more DAPs than OP insecticides by molar ratio. Chen et al. [22] confirmed the presence of DAPs and OP insecticides in produce and also measured loss of fenpropathrin and its degradate (3-PBA) in strawberries. DAPs have been observed in fresh fruit juices, and DAP production in juices fortified with OP insecticides has also been followed [16].

Oxidation is another hypothesized degradation route for some insecticides. For example, the first step in mammalian biological metabolism of OP insecticides is oxidation [24]. Insecticide oxidation has also been observed in red grape juice, water, and water fortified with the antioxidant quercetin [25]. In this study, quercetin and other components of grape juice were found to inhibit insecticide oxidation.

Degradation of insecticides in food and beverages is of importance for many reasons. Multiple studies have shown that the same compounds used as urinary metabolites are often produced in food before insecticide metabolism in the body [16,23,26]. Some insecticide degradates, such as DAPs, may be further degraded after ingestion [16]. However, other research concerning both DAPs and 3,5,6-trichloro-2-pyridinol (TCPy, a specific metabolite of chlorpyrifos and chlorpyrifos-methyl) in animal models suggests that these compounds are largely adsorbed by the body and then excreted unchanged in the urine [26]. If ingested insecticide degradates are excreted unchanged in urine, observation of these analytes would lead to overestimation of insecticide exposure.

Given neurodevelopmental dangers associated specifically with chlorpyrifos [27,28,29], this compound has been under particular scrutiny in the United States [30]. To direct related policy, we need full understanding of chlorpyrifos exposure and exposure assessment. Given this fact and the fact that chlorpyrifos is currently used on citrus crops [30], it is noteworthy that chlorpyrifos does not significantly degrade in orange juice. Therefore, it may be assumed that urinary chlorpyrifos metabolites such as 3,5,6-trichloro-2-pyridinol are effective BOEs for chlorpyrifos, supporting studies linking urinary TCPy concentrations to developmental effects in children [27,28,29].

Researchers have often assumed in the past that there is little to no toxicity from insecticide degradation product exposure. However, this assumption may not be the case. For example, there is evidence that 3-PBA is an antagonist of the human androgen receptor, potentially making it an endocrine disruptor [31]. Regardless of our current knowledge of insecticide degradate toxicity, there are currently few data on the adsorption, further metabolism, and potential toxicity of many insecticide degradates [16,32]. Therefore, it is difficult to know whether health effects parent insecticides, or if their degradates are being observed. Further research is needed to understand better the health effects of insecticide degradates.

The results of this study are important because they shed light on the degradation of insecticides in beverages, which in turn provides information exposure assessment of insecticides. All the pesticides demonstrated statistically significant degradation in at least one matrix. However, there was still a large fraction of parent insecticide left in all instances except diazinon in red wine. This result shows us that, while using urinary insecticide metabolites as insecticides BOEs may not be quantitatively accurate, they are still a good indication that parent insecticide exposure has likely occurred.

## 5. Conclusions

Statistically significant degradation of OP and pyrethroid insecticides is seen in several liquid beverage matrices. Our data support the hypothesis that loss of insecticides over time is due to degradation and not some other mechanism, such as adsorption onto the glass wall of the matrix container. These data support the contention that more research is needed regarding the exposure to insecticide degradation products and its relation to the use of urinary insecticide metabolites as biomarkers of exposure to insecticides.

## Figures and Tables

**Table 1:** Retention times of insecticides.

Insecticide	Retention Time (min)
diazinon	16.0
malathion	17.9
chlorpyrifos	18.2
permethrin	24.5
cyfluthrin	25.2
cypermethrin	25.5
deltamethrin	27.4

**Table 2:** Insecticide degradation results by GC-ECD. ^a^ Units of ^1^/_day_. ^b^ Standard error of rate constant in ^1^/_day_. ^c^ In days. Half-lives rendered meaningless due to the standard error being greater than the regression slope magnitude are reported as “n/a.” ^d^ Recovery of initial insecticide concentration after fortification, calculated using average concentration from day 0.

	Regression Slope ^a^	Std. Error ^b^	*p*-Value	Half Life ^c^	% Recovery ^d^
**Water**					
diazinon	1.99× 10^−2^	6.85× 10^−3^	0.0274	15.2	69.50%
malathion	6.75× 10^−3^	1.85× 10^−3^	0.0107	44.6	125.20%
chlorpyrifos	8.92× 10^−3^	3.30× 10^−3^	0.0355	33.8	77.40%
permethrin	3.70× 10^−3^	1.24× 10^−3^	0.0244	81.5	105.30%
cyfluthrin	6.75× 10^−3^	2.13× 10^−3^	0.1284	44.6	97.80%
cypermethrin	8.12× 10^−3^	8.58× 10^−4^	0.0001	37.1	104.00%
deltamethrin	6.13× 10^−3^	2.48× 10^−3^	0.0480	49.1	104.40%
**Grape Juice**					
diazinon	9.20× 10^−2^	1.69× 10^−2^	0.0016	3.3	76.30%
malathion	4.61× 10^−3^	2.82× 10^−3^	0.1535	65.3	137.10%
chlorpyrifos	1.62× 10^−2^	5.14× 10^−3^	0.0199	18.6	85.30%
permethrin	1.49× 10^−2^	1.97× 10^−3^	0.0003	20.2	90.80%
cyfluthrin	4.61× 10^−3^	2.65× 10^−3^	0.0002	65.3	80.50%
cypermethrin	1.80× 10^−2^	3.06× 10^−3^	0.0011	16.7	89.20%
deltamethrin	1.72× 10^−2^	5.28× 10^−3^	0.0174	17.5	73.60%
**Red Wine**					
diazinon	1.29× 10^−1^	1.14× 10^−2^	0.0003	2.3	40.70%
malathion	8.90× 10^−3^	3.86× 10^−3^	0.0606	33.8	111.40%
chlorpyrifos	1.25× 10^−2^	5.84× 10^−3^	0.0766	24.1	68.40%
permethrin	9.99× 10^−3^	2.78× 10^−3^	0.0114	30.1	106.90%
cyfluthrin	1.18× 10^−2^	3.57× 10^−3^	0.0161	25.5	105.10%
cypermethrin	1.04× 10^−2^	3.17× 10^−3^	0.0167	28.9	119.30%
deltamethrin	1.32× 10^−2^	4.02× 10^−3^	0.0170	n/a	109.90%
**Orange Juice**					
diazinon	2.34× 10^−2^	1.15× 10^−2^	0.0881	12.9	97.00%
malathion	6.33× 10^−3^	3.64× 10^−3^	0.1331	47.6	140.20%
chlorpyrifos	8.06× 10^−3^	6.89× 10^−3^	0.2865	37.3	93.00%
permethrin	6.97× 10^−4^	2.34× 10^−3^	0.7761	n/a	88.10%
cyfluthrin	3.20× 10^−3^	3.07× 10^−3^	0.3377	94.1	78.70%
cypermethrin	4.82× 10^−3^	1.62× 10^−3^	0.0250	62.5	82.10%
deltamethrin	-1.82× 10^−3^	3.74× 10^−3^	0.6442	n/a	69.60%
